# A combination of an anti-SLAMF6 antibody and ibrutinib efficiently abrogates expansion of chronic lymphocytic leukemia cells

**DOI:** 10.18632/oncotarget.8378

**Published:** 2016-03-25

**Authors:** Burcu Yigit, Peter J. Halibozek, Shih-Shih Chen, Michael S. O'Keeffe, Jon Arnason, David Avigan, Valter Gattei, Atul Bhan, Osman Cen, Richard Longnecker, Nicholas Chiorazzi, Ninghai Wang, Pablo Engel, Cox Terhorst

**Affiliations:** ^1^ Division of Immunology, Beth Israel Deaconess Medical Center, Harvard Medical School, Boston, MA, USA; ^2^ Karches Center for Chronic Lymphocytic Leukemia Research, The Feinstein Institute for Medical Research, Manhasset, NY, USA; ^3^ Division of Hematology/Oncology, Beth Israel Deaconess Medical Center, Harvard Medical School, Boston, MA, USA; ^4^ Clinical and Experimental Onco-Hematology Unit, Centro di Riferimento Oncologico, I.R.C.C.S., Aviano, Italy; ^5^ Department of Pathology, Massachusetts General Hospital, Harvard Medical School, Boston, MA, USA; ^6^ Department of Microbiology and Immunology, Northwestern University Feinberg School of Medicine, Chicago, IL, USA; ^7^ Immunology Unit, Department of Cell Biology, Immunology and Neurosciences, Medical School, University of Barcelona, Barcelona, Spain

**Keywords:** CLL, TCL1-192, SLAMF6, BCR, ibrutinib

## Abstract

The signaling lymphocyte activation molecule family [SLAMF] of cell surface receptors partakes in both the development of several immunocyte lineages and innate and adaptive immune responses in humans and mice. For instance, the homophilic molecule SLAMF6 (CD352) is in part involved in natural killer T cell development, but also modulates T follicular helper cell and germinal B cell interactions. Here we report that upon transplantation of a well-defined aggressive murine B220^+^CD5^+^ Chronic Lymphocytic Leukemia (CLL) cell clone, TCL1-192, into SCID mice one injection of a monoclonal antibody directed against SLAMF6 (αSlamf6) abrogates tumor progression in the spleen, bone marrow and blood. Similarly, progression of a murine B cell lymphoma, LMP2A/λMyc, was also eliminated by αSlamf6. But, surprisingly, αSLAMF6 neither eliminated TCL1-192 nor LMP2A/λMyc cells, which resided in the peritoneal cavity or omentum. This appeared to be dependent upon the tumor environment, which affected the frequency of sub-populations of the TCL1-192 clone or the inability of peritoneal macrophages to induce Antibody Dependent Cellular Cytotoxicity (ADCC). However, co-administering αSlamf6 with the Bruton tyrosine kinase (Btk) inhibitor, ibrutinib, synergized to efficiently eliminate the tumor cells in the spleen, bone marrow, liver and the peritoneal cavity. Because an anti-human SLAMF6 mAb efficiently killed human CLL cells *in vitro* and *in vivo*, we propose that a combination of αSlamf6 with ibrutinib should be considered as a novel therapeutic approach for CLL and other B cell tumors.

## INTRODUCTION

Chronic lymphocytic leukemia (CLL) is the most common B cell leukemia in adults and is characterized by the accumulation of CD19^+^CD5^+^CD23^+^ B cells in the bone marrow, blood and secondary lymphoid organs [1]. Survival and proliferation of these tumors depend on two main factors: the tumor microenvironment and B cell receptor (BCR) signaling [2, 3]. Within CLL “proliferation centers”, tumor cells interact with monocyte derived nurse-like cells (NLCs) [2–6], which provide survival signals via secretion of chemokines and cytokines [7, 8], or interacting cell surface receptor-ligand structures. The latter include CD74/MIF, CD84 (SLAMF5) and CD150 (SLAMF1) [9–11].

Here we evaluate the efficacy of monoclonal antibodies directed against mouse and human SLAMF6 (CD352) in CLL prognosis. SLAMF6 [12] is a homophilic SLAMF receptor [13–22], which plays a key role in the interactions between T follicular helper (TFH) cells and Germinal Center B (GCB) cells [19, 23–32]. To study the effect of anti-SLAMF6 on tumor progression we use the aggressive transplantable murine CLL clone TCL1-192 and the B cell lymphoma LMP2A/λMyc [33–35] into SCID or Rag1^−/−^ mice, respectively. The murine TCL1-192 clone expresses a B cell receptor with a single IGHV-D-J arrangement, which is specific for phosphatidyl choline [PtC], has many characteristics in common with human CLL cells [33]. As the transfer of TCL1-192 into SCID mice leads to an aggressive disease progression within 5-6 weeks, this offers a very useful platform to identify relevant potential therapeutic targets [33].

The data indicate that removal of the tumor cells by a mouse anti-mouse Slamf6 (αSlamf6) antibody (13G3) [26], see Materials and Methods) relies on antibody dependent cell-mediated cytotoxicity (ADCC) and co-stimulation of B cell receptor (BCR) signaling, which is of importance to progression of CLL. Co-administering αSlamf6 with the Bruton tyrosine kinase (Btk) inhibitor ibrutinib has a synergistic effect on treatment of the tumors. Furthermore, we provide evidence that mouse anti-human SLAMF6 (αhSLAMF6) antibody is efficient in *in vitro* and *in vivo* killing of two CLL cell lines MEC-1 and OSU-CLL [36, 37].

## RESULTS

### Administering αSlamf6 prevents expansion of TCL1-192 cells in the spleen and blood, but not in the peritoneal cavity

We first determined that surface expression of SLAMF receptors by TCL1-192 cells [33] is comparable to SLAMF surface expression by patient-derived human CLL cells and the CLL cell lines MEC1 and OSU-CLL (Supplementary Figure S1 and S2). Consistent with its high level of expression by B lineage cells [38], this SLAMF6 is found on the surface of freshly isolated human CLL cells (Supplementary Figure S1C) or frozen patient cells (Supplementary Figure S2). Whereas SLAMF6 expression varies somewhat between CLL cells from different patients, SLAMF1 and SLAMF7 expression differs more between individual patients (Supplementary Figure S2). Similar to its relative expression by mouse B cells, (www.immgen.org) [26], Slamf6 is highly expressed on the surface of TCL1-192 cells. Surprisingly, the level of expression of Slamf6 on the surface of TCL1-192 cells in the peritoneal cavity was twice that on cells isolated from the blood or spleen (MFI P: 23739, B: 13279, S: 14384) (Supplementary Figure S1).

**Figure 1:**
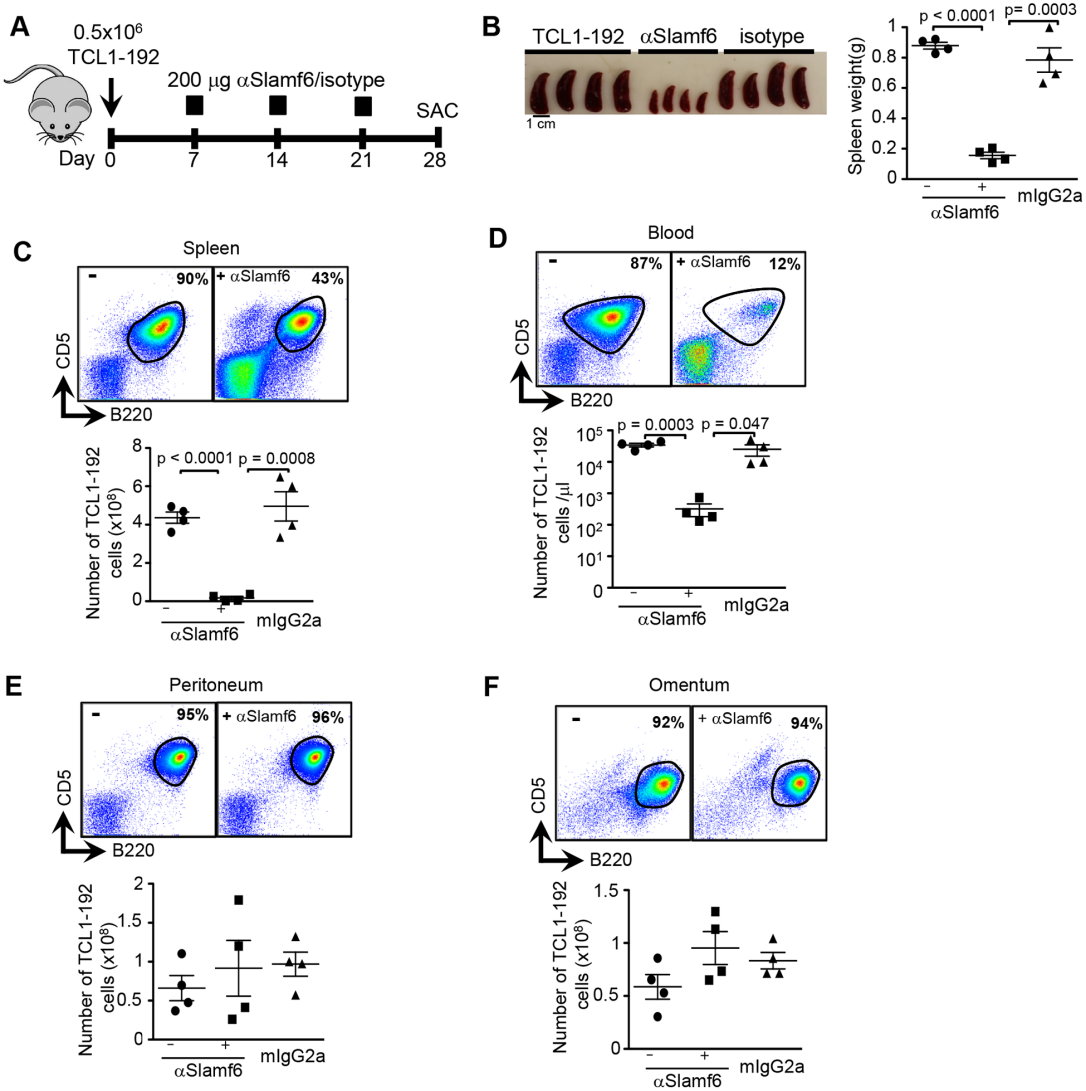
Anti-Slamf6 prevents TCL1-192 expansion in the spleen and blood, but not in the peritoneal cavity, of SCID mice **A.** Schematic outline of the prevention experiment. TCL1-192 cells were injected on d0 and 200μg mouse αSlamf6 (13G3) or a mouse IgG2a isotype control was injected *i.p.* into SCID mice on day 7, 14 and 21. Mice were sacrificed on day 28. **B.** Spleen size and weight at day 28. Administering αSlamf6 vs IgG2a isotype caused a 5.0- fold reduction (0.15 ± 0.02 vs. 0.78 ± 0.08 g; *p = 0.0003*) or 5.8-fold reductions comparing αSlamf6 *vs.* no antibody (0.15 ± 0.02 vs. 0.87 ± 0.02 g; *p < 0.0001*). **C.** A 26-fold reduction of the number of TCL1-192 cells was detected in the spleen of αSlamf6-injected vs. non-injected (1.7 ± 0.8 × 10^7^ vs. 4.4 ± 0.3 × 10^8^ *p < 0.0001*); or 29-fold in αSlamf6-injected vs. isotype-injected (1.7 ± 0.8 × 10^7^ vs. 4.9 ± 0.7 × 10^8^; *p = 0.0008*) SCID mice. Cells were gated on viable, DAPI^−^, B220^+^CD5^+^ cells. Counting beads were used to determine the number of TCL1-192 cells per μl of blood. **D.** A 113-fold reduction of TCL1-192 cells in the blood of αSlamf6-injected vs. non-injected mice was found (0.3 ± 0.1 × 10^3^ *vs.* 3.4 ± 0.4 × 10^4^ per μl blood; *p = 0.0003*); 100-fold in αSlamf6-injected vs. isotype-injected mice (0.3 ± 0.1 × 10^3^ *vs.* 3 ± 1.1 × 10^4^ per μl blood; *p = 0.047)*. **E.** Number of TCL1-192 cells in the peritoneal cavity: αSlamf6-injected vs. non-injected (9.38 ± 3.6 × 10^6^ *vs.* 5.8 ± 2.3 × 10^6^) or αSlamf6-injected vs. isotype-injected (9.38 ± 3.6 × 10^6^*vs.* 1 ± 0.1 × 10^7^). **F.** Number of TCL1-192 cells in the omentum: αSlamf6-injected vs. non-injected (9.5 ± 1.55 × 10^6^ *vs.* 5.9 ± 1.2 × 10^6^ or αSlamf6-injected *vs.* isotype-injected (9.5 ± 1.55 × 10^6^ *vs.* 8.3 ± 0.7 × 10^6^). Results are representative of at least 3 independent experiments.

To assess the efficacy of αSlamf6 in preventing expansion of the mouse CLL cells, αSlamf6 IgG2a was administered on day 7, 14 and 21 post-transplant of the TCL1-192 cells into SCID mice (Figure 1A). Prior to these experiments we had determined that one week after injecting 0.5 × 10^6^ TCL1-192 cells *i.p.* into a SCID mouse, the cells primarily reside in the peritoneal cavity, but that at day 28, the tumor cells have expanded and are found in the peritoneal cavity [~1 × 10^8^], spleen [~4 × 10^8^], and blood [~10^5^/μl] (data not shown). Importantly, in a previous study a similar distribution of TCL1-192 cells was found regardless of whether the tumor cells were injected *i.v*. or *i.p.* [33].

At day 28 the spleen size of αSlamf6-treated mice was 20% of the spleen size of recipients of isotype-control mice or of mice that had not received antibody (Figure 1B). More importantly, the number of leukemic cells in the spleen of recipients of αSlamf6 injected mice was 26 fold reduced (Figure 1C). TCL1-192 cells were virtually absent in the blood of αSlamf6-injected mice compared to the control mice (Figure 1D). Surprisingly, αSlamf6 did not affect the number of tumor cells in the peritoneal cavity (Figure 1E) or in the omentum, a well-known reservoir for B1a cells [39] (Figure 1F). On day 28 expression of Slamf6 by the leukemic cells in the peritoneal cavity, blood and spleen from all groups was comparable (Supplementary Figure S3A).

Together the data show that, three injections of αSlamf6 eliminated TCL1-192 cells in the spleen and blood of the recipient mice, but not in the peritoneal cavity.

### Administering αSlamf6 reduced the number of LMP2A/λMyc B cell lymphomas in Rag-1^−/−^ mice

**Figure 2:**
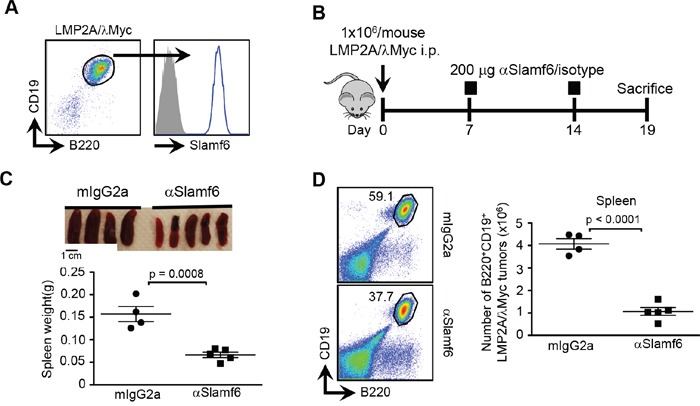
Anti-Slamf6 reduces tumor burden in LMP2A/λMyc bearing Rag1^−/−^ mice **A.** Expression of Slamf6 on CD19^+^CD20^+^ LMP2A/lMyc cells. **B.** Schematic outline of the experiment. Rag1^−/−^ mice were i.p. injected with 1 × 10^6^ cells and injected *i.p.* with 200 μg αSlamf6 or isotype control on day 7 and 14. Mice were sacrificed on day 19. **C.** Differences in spleen size and weight on day 19. A 3-fold reduction was observed in spleen weight of mice injected with αSlamf6 compared to isotype-injected group (0.066 ± 0.005 vs. 0.15 ± 0.01; p = 0.0008). **D.** Percentage of CD19^+^CD20^+^ LMP2A/λMyc tumors and absolute cell numbers in the spleen are shown. A 4-fold reduction in tumor burden was observed in αSlamf6-injected group (1.05 ± 0.17 × 10^6^ vs. 4.07 ± 0.22 × 10^6^; p < 0.0001). Representative of 2 independent experiments.

To evaluate whether αSlamf6 would also effectively remove an unrelated CD19^+^B220^+^ murine B cell lymphoma, LMP2A/λMyc [35], which expresses Slamf6 (Figure 2A), on day 7 and 14 after *i.p* injection of LMP2A/λMyc [1 × 10^6^ cells/mouse] into *Rag1^−/−^* mice, 200μg/mouse αSlamf6 or isotype control was administered (Figure 2B). On day 19 post-transplant αSlamf6 treated mice had significantly smaller spleens (Figure 2C) and less tumor cells than did control mice (Figure 2C and 2D). Thus, like in the case of the TCL1-192 CLL cells, αSlamf6 also reduces the number LMP2A/λMyc lymphoma cells.

### Treatment with αSlamf6 after expansion of TCL1-192 cells in SCID mice

**Figure 3:**
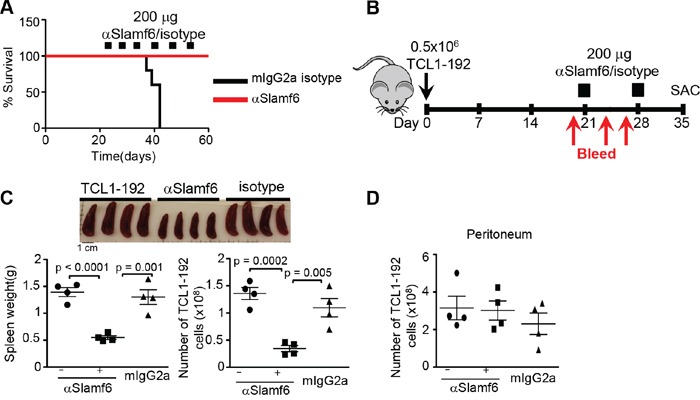
Treatment with αSlamf6 of TCL1-192 in the spleen, but not in the peritoneal cavity of SCID mice **A.** Three weeks after transplanting TCL1-192 cells, SCID mice were injected with αSlamf6 or isotype mIgG2a once a week (Squares indicate the number of injections). Mice were monitored everyday. Whereas isotype treated mice died between 35-42 days, αSlamf6 injected mice were still alive on day 60, when the experiment ended (n = 5 mice each group). **B.** Schematic outline of the experiment indicating the timeline of i.p. injection of TCL1-192 cells and *i.p*. injections of 200 μg αSlamf6 (13G3) or the IgG2a isotype control on day 21 and 28. Mice were monitored by bleeding on days 21, 24 and 27 and were sacrificed on day 35. **C.** A 2.5-fold reduction in spleen size was caused in αSlamf6 vs. isotype treated mice (0.55 ± 0.03 vs. 1.4 ± 0.08g; *p = 0.0018)* or 2.4-fold vs. untreated mice (1.303 ± 0.13 g; *p < 0.0001*) on day 35. The number of TCL1-192 cells was 3-fold reduced in αSlamf6 vs. isotype treated mice (3.4 ± 0.6 × 10^8^ vs. 1.1 ± 0.1 × 10^9^; *p = 0.005*) or 4-fold vs. untreated mice (3.4 ± 0.6 × 10^8^ vs. 1.36 ± 0.1 × 10^9^ *p = 0.0002*). **D.** The number of TCL1-192 cells in the peritoneum of αSlamf6 injected and isotype or non-injected SCID mice (3 ± 0.5 × 10^8^ vs. 2.3 ± 0.5 × 10^8^ and 3.1 ± 0.6 × 10^8^ *p = 0.8* and *p = 0.4*). The data are representative of 3 independent experiments. Results are representative of 4 independent experiments.

Next, we employed a “treatment protocol” to assess whether αSlamf6 would affect survival of SCID mice in which TCL1-192 cells had expanded for three weeks after transplant and antibodies were subsequently administered *i.p.* once a week (Figure 3A). While after 6 injections of αSlamf6 recipient mice were alive at day 60, the mice that had received control mIgG2a died between 35-42 days (Figure 3A).

In order to analyze the difference in leukemic burden between αSlamf6 and mIgG2a injected mice, αSlamf6 was administered only twice, *i.e.* on day 21 and day 28 after transplanting the TCL1-192 cells (outlined in Figure 3B). Mice were sacrificed on day 35 when the control group was moribund. On day 35, the spleen size of αSlamf6 treated mice was reduced 2.5 fold (Figure 3C) and the massive tumor infiltrate in the liver was absent in the αSlamf6 treated group compared to the two control groups (Supplementary Figure S4A). The total number of TCL1-192 cells in the spleen of αSlamf6 treated mice was significantly less than that of the control mice (Figure 3C). As in the prevention experiments, the leukemic cells from the peritoneal cavity were not affected by the monoclonal antibody (Figure 3D). The outcomes of these experiments indicate that treatment of existing tumors with two injections of αSlamf6 effectively eliminates TCL1-192 cells in the spleen, but not in the peritoneum.

### Anti-Slamf6 eliminates TCL1-192 cells in the blood of transplanted SCID mice

**Figure 4:**
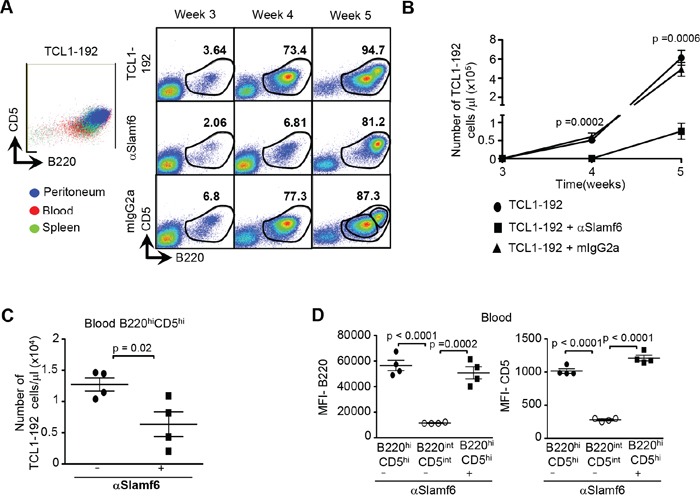
The number of TCL1-192 cells is reduced in the blood of SCID mice upon treatment with αSlamf6 Schematic outline of the experiment is described in Figure 3B. **A. Left Panel:** Representative flow cytometry plot showing the overlay of B220^+^CD5^+^ cells from the peritoneum, blood and spleen. While TCL1-192 cells from the peritoneum, spleen and blood express comparable levels of high B220 and CD5; the blood contains a second population that expresses lower levels of B220 and CD5 (named B220^int^CD5^int^). **Right Panel:** Percentage of B220^+^CD5^+^ TCL1-192 cells in the blood of SCID mice at weeks 3, 4 and 5 in non-injected and αSlamf6 or mIgG2a injected groups. Representative gating for the two sub-populations (B220^int^CD5^int^ and B220^hi^CD5^hi^) is depicted in the lower right corner for the isotype control group. **B.** On day 28, the total B220^+^CD5^+^ cell number per μl blood is a 100-fold less in αSlamf6-injected vs. mIgG2a injected mice (per μl blood: 0.6 ± 0.1 × 10^3^ vs. 6 ± 1 × 10^4^; *p = 0.001*); or 85-fold less as compared to the non-injected group (0.6 ± 0.1 × 10^3^ vs 5.1 ± 0.6 × 10^4^; p = *0.0002*). Although cells keep expanding by day 35, αSlamf6 injected group still has significantly less leukemic burden compared to mIgG2a injected (per μl blood: 7.5 ± 2.1 × 10^4^ vs. 4.9 ± 0.7 × 10^5^; *p = 0.0015*) vs. non-injected group (6.1 ± 0.7 × 10^5^; *p = 0.0006*). The data are representative of 3 independent experiments. **C.** Number of B220^hi^CD5^hi^ leukemic cells in the blood of αSlamf6 treated mice is less than that in non-treated mice (0.63 ± 0.19 × 10^5^ vs. 1.2 ± 0.1 × 10^5^; p = 0.02). **D.** Mean Fluorescence Intensity (MFI) values of B220 and CD5 ex pression on B220^hi^CD5^hi^ and B220^int^CD5^int^ populations of the non-treated group at week 5. Results are representative of 4 independent experiments.

One week after the first injection of αSlamf6, *i.e.* on day 28, the number of B220^+^CD5^+^ TCL1-192 cells in the blood (Figure 4A Right panel and 4B) was lower than in control mice. However, at day 35, a week after the second injection, the number of TCL1-192 cells in the blood had increased in αSlamf6-injected mice, although it remained significantly lower than in the control mice (Figure 4A–4C). This raises the possibility that on day 28 before the second αSlamf6 injection, αSlamf6-IgG from the first injection still occupied the receptor, which rendered the second injection of αSlamf6 ineffective (Figure 3B). This is indeed the case, because the Slamf6 receptor on the surface of the blood TCL1-192 cells was not accessible by a PE-conjugated αSlamf6 on 3 days after the first antibody injection [on day 24] and was only partially accessible on day 27, six days after the first injection (Supplementary Figure S3B). This is consistent with the half-life of mouse IgG2a of 6-8 days [40]. The data indicate that only the first injection of αSlamf6 was effective.

### Distinct responses to αSlamf6 by the B220^hi^CD5^hi^ and B220^int^CD5^int^ TCL1-192 cell subsets

The dependence of the αSlamf6 antibody treatment on the location of the tumor cells prompted us to compare the key properties of the TCL1-192 cells in peritoneum, blood and spleen. Thirty-five days after transplant TCL1-192 cells in the peritoneum and spleen consisted primarily of a B220^hi^CD5^hi^ sub-population, whereas both B220^int^CD5^int^ and B220^hi^CD5^hi^ cells were found in the blood (Figure 4A Left Panel). As the B220^int^ CD5^int^ TCL1-192 cells are absent from the blood of αSlamf6 treated mice, this sub-population appears to have been eliminated by the antibody. By contrast, the B220^hi^CD5^hi^ TCL1-192 subpopulation appears resistant to the αSlamf6 treatment (Figure 4A and 4D).

**Figure 5:**
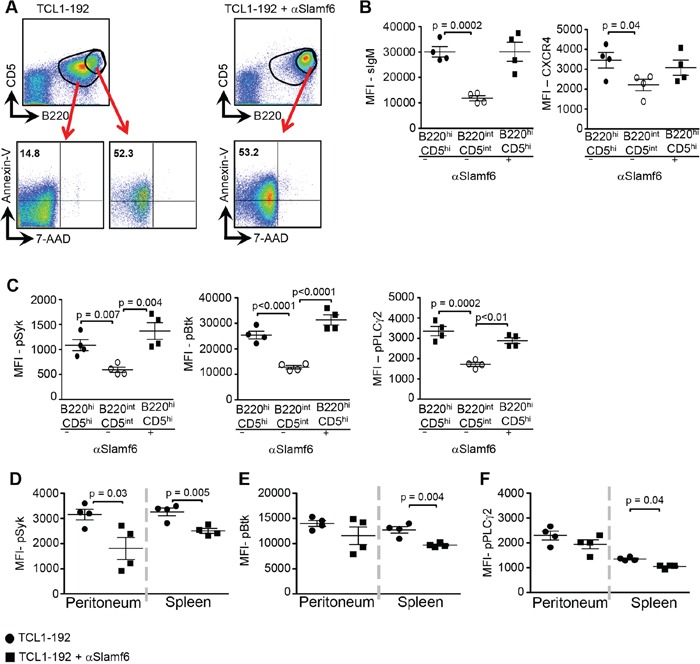
Differential αSlamf6-dependent signaling in B220^hi^CD5^hi^ and B220^int^CD5^int^ TCL1-192 CLL cells in the blood, spleen and peritoneum Schematic outline of the experiment is described in Figure 3B. **A.** Comparison of total B220^+^CD5^+^ cells in blood of the non-treated group (−) to its B220^hi^CD5^hi^ subpopulation and B220^hi^CD5^hi^ TCL1-192 cells in the αSlamf6-treated (+) mice by Annexin-V/7-AAD staining. **B.** MFI values of surface IgM (sIgM) and CXCR4 expression on B220^hi^CD5^hi^ and B220^int^CD5^int^ TCL1-192 cells in the blood of αSlamf6-treated (+) and non-treated (−) mice. **C.** Proximal BCR signaling in B220^hi^CD5^hi^ and B220^int^CD5^int^ populations in the blood of αSlamf6-treated (+) and non-treated (−) mice was assessed by intracellular staining of pSyk, pBtk and pPLCγ2 and measured by flow cytometry. Representative of three experiments. **D-F.** Expression of pSyk, pBtk and pPLCγ2 in spleen and peritoneum. Results are representative of 4 independent experiments.

These results prompted us to compare the state of activation, viability and signaling of the subpopulations in the blood of the treated and non-treated mice. Approximately half of the B220^hi^CD5^hi^ TCL1-192 subpopulation appears to be early apoptotic, as judged by Annexin-V/7-AAD staining (Figure 5A and Supplementary Figure S5A). By contrast, the B220^int^CD5^int^ population, which is eliminated by αSlamf6, is Annexin-V/7-AAD negative (Figure 5A).

The resistance of the B220^hi^CD5^hi^ TCL1-192 sub-population in the blood to αSlamf6 treatment also coincides with high expression of the BCR (Figure 5B). BCR proximal signaling, as judged by pSyk, pBtk and pPLCγ2, is also higher in the B220^hi^CD5^hi^ population than in the B220^int^CD5^int^ population of TCL1-192 cells (Figure 5C). Expression of the chemokine receptor CXCR4 was significantly higher in the B220^hi^CD5^hi^ subset than in the B220^int^CD5^int^ subset (Figure 5B, Right Panel). Overall, the B220^hi^CD5^hi^ population presents a mixture of cells, while some are undergoing apoptosis, remaining cells are on their way to migrating to secondary lymphoid organs, as suggested by their high BCR and CXCR4 expression levels [41].

The levels of pSyk, pBtk and pPLCγ2 were significantly lower in splenic TCL1-192 cells of αSlamf6 treated mice compared to those in non-injected SCID mice (Figure 5D–5F and Supplementary Figure S5B). Interestingly, BCR signaling by peritoneal cavity TCL1-192 cells differed from signaling by splenic tumor cells. Together, these findings suggest that the αSlamf6 antibody impacts the leukemic cells differently; most likely depending on the microenvironment in which the cells reside.

### Administering αSlamf6 down-regulates proximal BCR signaling and induces ADCC

**Figure 6:**
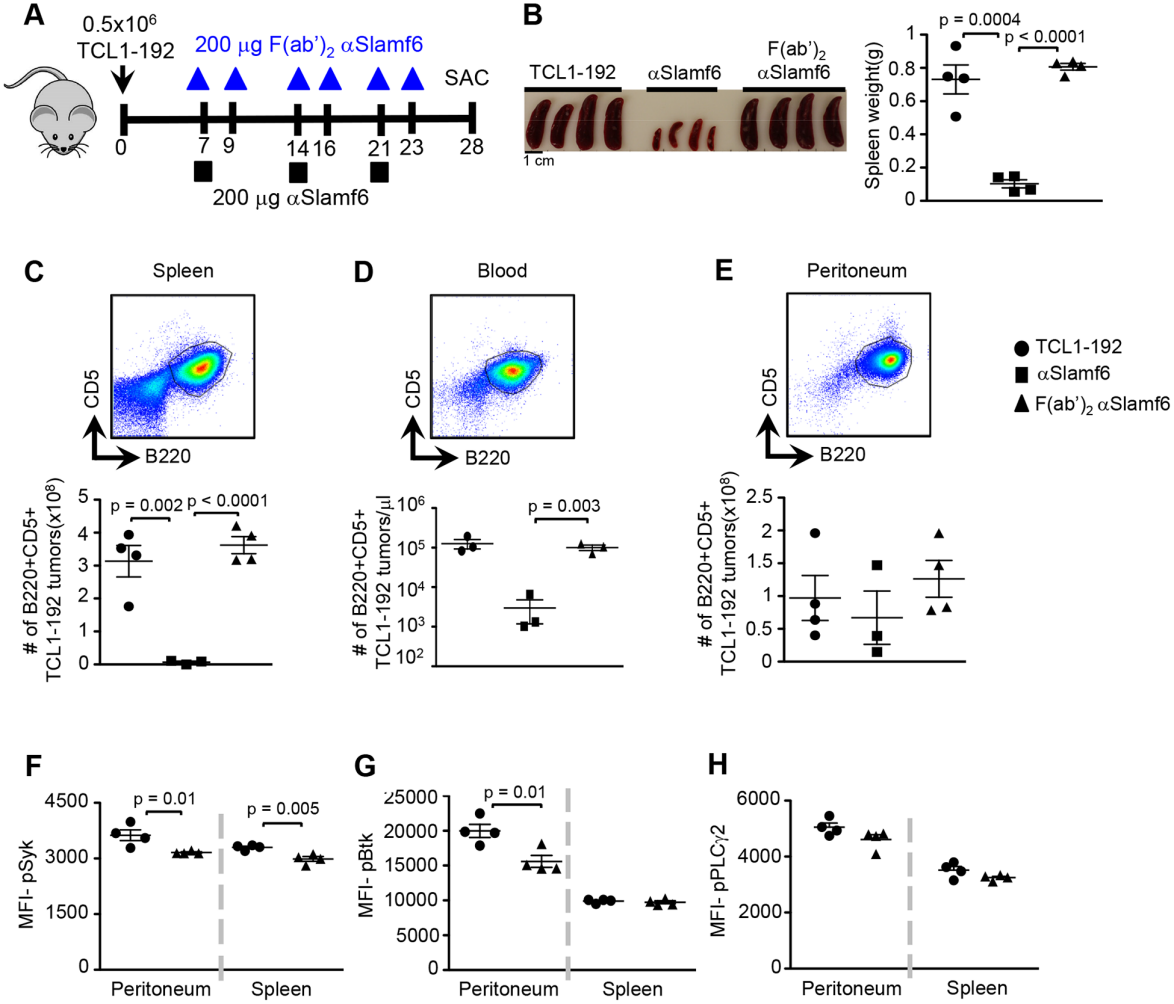
Administering αSlamf6 F(ab')_2_ fragments does not reduce the number of TCL1-192 cells F(ab')_2_ αSlamf6 was generated using Pierce F(ab')_2_ preparation kit. **A.** Outline of the experiment. TCL1-192 cells were i.p. injected on d0. On d7, d14 and d21, 200 μg αSlamf6 (13G3) was i.p. injected, or on d7, d9, d14, d16, d21 and d23, 200 μg F(ab')_2_ αSlamf6 was injected. Mice were sacrificed on d28. **B.** Differences in spleen size and weight in non-injected, αSlamf6 or F(ab')_2_ αSlamf6 injected SCID mice on d28. **C-E.** Representative staining of B220+CD5+ from non-injected mice and number of B220+CD5+ cells in SCID mice after αSlamf6, F(ab')_2_ αSlamf6 injected were compared to those of non-injected mice in spleen, blood and peritoneum. Next, levels of pSyk **F.**, pBtk **G.** and pPLCγ2 **H.** in TCL1-192 cells isolated on d28 from non-injected and F(ab')_2_ αSlamf6 injected mice were compared. Representative of 2 independent experiments with n = 9 mice total for F(ab')_2_ αSlamf6 injected group. P values are as shown.

It is likely that the removal of leukemic cells in the blood involves αSlamf6-induced signaling in the TCL1-192 cells, as well as cell mediated cytotoxicity (ADCC) by macrophages or NK cells [42]. To further assess the possibility that administering αSlamf6 instigates co-stimulatory signaling networks in TCL1-192 cells, six doses of 200 μg/mouse of F(ab')_2_ αSlamf6 were injected (outlined in Figure 6A). Although the leukemic burden was not affected (Figure 6B–6E), probably due to the short half life of the F(ab')_2_ fragments *in vivo*, BCR signaling in spleen and peritoneum was observed (Figure 6F–6H). In sum, the BCR signaling of TCL1-192 cells, which is induced by endogenous PtC [33], is affected by treatment of the CLL bearing SCID mice with αSlamf6. It would therefore appear that αSlamf6 regulates both BCR signaling through the Slamf6 receptor and efficiently induces ADCC.

### Absence of CLL cells in the peritoneal cavity after co-administering the BTK inhibitor ibrutinib

Although αSlamf6 antibody by itself is very effective in targeting the leukemic cells in blood, spleen and liver, it did not affect cells that reside in niches, e.g. the peritoneal cavity and omentum. Because macrophages and monocytes of the mouse peritoneal cavity are often found in lymphoid aggregates with T cells and CD5^+^B220^+^ B1 cells, which are almost absent in lymphoid tissues, we reasoned that the CLL cells resided in niches, which prevent efficient killing by αSlamf6 or other monoclonal antibodies [42]. Because in CLL patients the BTK inhibitor, ibrutinib, is known to release the tumor cells from their niches into the blood [43], we combined αSlamf6 treatment with administering ibrutinib.

**Figure 7:**
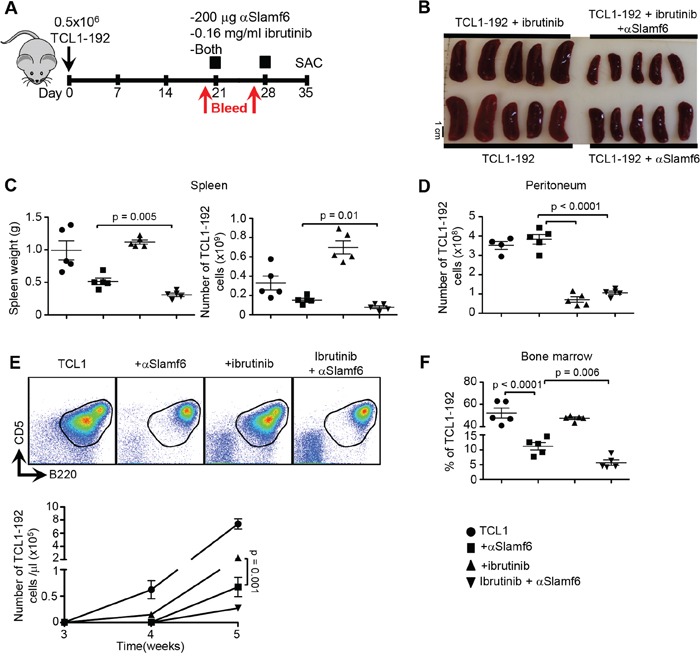
Co-administering αSlamf6 with ibrutinib significantly reduces the number of TCL1-192 cells in the peritoneal cavity **A.** Outline of the experiment. TCL1-192 cells were *i.p*. injected on d0. On d21, group of mice were either *i.p*. injected 200 μg αSlamf6 (13G3), given 0.16 mg/ml ibrutinib in drinking water or was given both. Mice on ibrutinib received the drug until sacrifice. Mice received another injection of αSlamf6 on d28. Mice were sacrificed on d28. **B.** Differences in spleen size is as shown. **C.** Mice treated with a combination of αSlamf6 and ibrutinib had significantly smaller spleens and leukemic burden compared to αSlamf6-treated mice (weight: 0.31 ± 0.02 vs. 0.51 ± 0.04 g; *p = 0.005* and a two-fold smaller number of TCL1-192 cells: 0.78 ×10^8^ ± 1.54 × 10^7^ vs. 1.53 × 10^8^ ± 1.76 × 10^7^; *p = 0.01*) on day 35. **D.** Relative numbers of TCL1-192 cells in the peritoneal cavity. BTK inhibitor resulted in 5.4 fold reduction in leukemic burden in peritoneal cavity compared to the control or αSlamf6 treated group alone (7.07 ± 1.43 × 10^7^ vs. 3.82 ± 0.24 × 10^8^; p < 0.0001). **E.** Representative FACS plots of B220^+^CD5^+^ in different groups and the exact cell numbers are as shown in the graph below. Ibrutinib alone treated mice resulted in 3-fold lower leukemic burden in blood compared to non-treated mice (per μl blood: 2.2 × 10^5^ ± 2.9 × 10^4^ vs. 7.3 × 10^5^ ± 7.7 × 10^4^; *p = 0.0003*). However, αSlamf6 alone treated mice had significantly less TCL1-192 burden compared to ibrutinib alone group (0.67 × 10^5^ ± 1.8 × 10^4^; *p = 0.0001*). Difference in leukemic burden between αSlamf6 alone and ibrutinib/αSlamf6 treated group did not reach statistical significance (0.67 × 10^5^ ± 1.8 × 10^4^ vs. 0.27 × 10^5^ ± 0.5 × 10^4^; *p = 0.06*). **F.** Percentage of TCL1-192 cells in the bone marrow. Anti-Slamf6 resulted in 4.6- fold reduction in percentage of TCL1-192 cells in bone marrow compared to non-treated group (52.2 ± 4.4 vs. 11.2 ± 1.2%; *p < 0.0001*). When compared, αSlamf6 in combination with ibrutinib resulted in a further reduction (2 fold) compared to αSlamf6 treatment alone (11.2 ± 1.2 vs. 5.6 ± 0.8%; *p = 0.006*). Representative of 2 independent experiments is shown. P values are as shown.

Using the treatment protocol (Figure 3B), SCID mice injected with TCL1-192 were given 200 μg/mouse αSlamf6 and 25 mg/kg/day ibrutinib in the drinking water on day 21 (Figure 7A). Ibrutinib was kept in the drinking water until sacrifice and αSlamf6 was injected again on day 28. The number of leukemic cells in the blood was determined on day 20 and 27 (Figure 7A).

The spleen size and number of TCL1-192 cells was reduced 2-fold in αSlamf6 / ibrutinib-treated mice as compared to the number of splenocytes in animals that had been treated with αSlamf6 alone (Figure 7B and 7C). As predicted, ibrutinib alone or αSlamf6 / ibrutinib efficiently eliminated TCL1-192 cells in the peritoneal cavity (Figure 7D).

Treatment with αSlamf6 / ibrutinib or αSlamf6 reduced the number of TCL1-192 cells by 95% and 90%, respectively. Ibrutinib-treatment alone reduced the number of TCL1-192 cells by 70% (Figure 7E). As in Figure 4A, the Slamf6 resulted in the loss of B220^int^CD5^int^ tumor cells, while ibrutinib did not differentially affect either sub-population.

The reduction of tumor cells in the bone marrow of αSlamf6 / ibrutinib treated animals was ten-fold, while αSlamf6 reduced the percentage of TCL1-192 cells only 4.6 fold (Figure 7F). H&E staining indicated a complete absence of leukemic cells in the liver of αSlamf6 / ibrutinib-treated mice as compared to either αSlamf6 or ibrutinib alone (Supplementary Figure S4B).

To further show the efficacy of αSlamf6 and ibrutinib treatment on TCL1-192 cells, we employed *in vitro* assays using both splenic and peritoneal cells and measured viability (AnnexinV/7AAD negative) after 72 hours of culturing with ibrutinib, αSlamf6 or both (Supplementary Figure S4C). In splenic TCL1-192 cells, ibrutinib or αSlamf6 alone led to a significant reduction in viability, and it was further reduced when the two compounds were combined. Interestingly, peritoneal cells were not affected by either treatment *in vitro*, suggesting that they are indeed more resistant to killing compared to splenic cells. This also indirectly suggests that *in vivo*, ibrutinib does remove the cells from the niche, rather than affecting their survival within the niche.

Taken together, the outcomes of these studies indicate that αSlamf6 and ibrutinib synergize in decreasing the overall leukemic burden.

### Anti-human SLAMF6 reduces the number of human CLL cells

**Figure 8:**
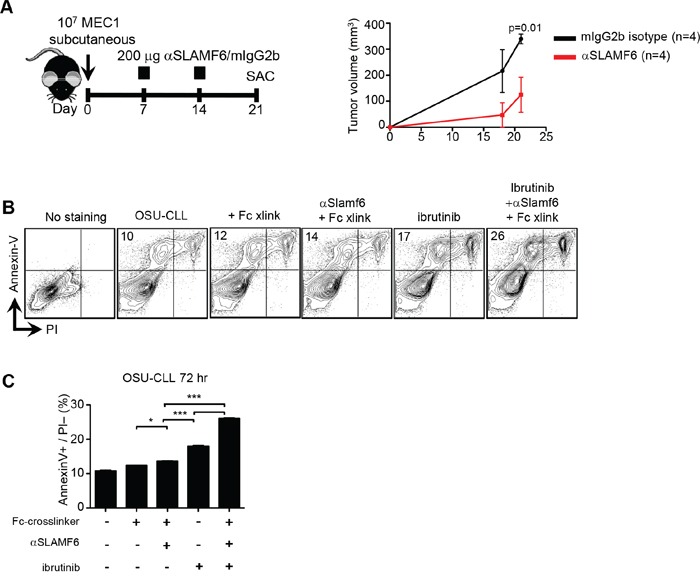
Anti-human SLAMF6 affects progression of human MEC-1 and OSU CLL cells *in vitro* and *in vivo* **A.** 10^7^ MEC-1 cells were subcutaneously injected into Rag2^−/−^γc^−/−^ mice. 200μg/ml mouse αhuman-SLAMF6 or isotype mIgG2b was injected *i.p.* on day 7 and 14 (**Left Panel**). Tumor volume was determined by measuring 3 diameters at indicated time points (**Right Panel**). **B-C.** OSU-CLL cells were cultured *in vitro* in the presence of 50 μg/ml Fc-crosslinker alone, 10 μg/ml mouse αhSLAMF6 and Fc-crosslinker, 0.5 μM ibrutinib or in combination for 72 hours and apoptosis was measured by Annexin-V+/PI-. **B.** The plots demonstrate the representative gating strategy for AnnexinV/PI staining. Results are representative of 3 independent experiments. (*: p < 0.05, **: p < 0.01, ***: p < 0.005)

In order to support the findings with the murine TCL-1 model, we assessed responses to αhSLAMF6 of the human MEC-1 and OSU-CLL cell lines, which highly express SLAMF6 (Supplementary Figure S1). First, we transplanted 10^7^ MEC-1 cells subcutaneously into [Rag x γc]^−/−^ mice [44] before administering αhSLAMF6 or an isotype control (mIgG2b) on days 7 and 14 (Figure 8A).

Twenty-one days after transplanting the MEC-1 cells, the volume of subcutaneous tumors in αhSLAMF6-injected mice was 3.5 times smaller than that in isotype control mice (Figure 8A). This difference was already indicated on day 18 without reaching statistical significance. Because the [Rag x γc]^−/−^ mice do not have NK cells, killing of MEC-1 tumors might be caused by macrophages via ADCC and possibly by αSLAMF6-induced signaling.

We also tested the effect of the αhSLAMF6 antibody in combination with ibrutinib on apoptosis of *in vitro* cultured human OSU-CLL cells [37]. The combination of ibrutinib with αhSLAMF6 resulted in a higher percentage of Annexin-V^+^ cells (Figure 8B and 8C), once again suggesting a synergistic effect of the two agents on human CLL cell survival.

## DISCUSSION

The pathogenesis of CLL is in part driven by signaling of the BCR in response to a restricted set of auto-antigens, which might function both during precursor cell initiation and clonal progression. Disease aggressiveness has been correlated with the cell surface density and the kinetics of membrane microdomain formation of the BCR and its signaling networks [45, 46]. Whereas clinical trials with small molecules that target BCR initiated signaling, *e.g.* inhibitors of Syk [47], PI3K [48] and Btk [49], have been successful, monoclonal antibodies remain promising as therapeutic targets. Here we show that αSlamf6 efficiently eliminates the murine CLL clone TCL1-192, which after transplantation into SCID mice resides in the spleen and blood. We have chosen this TCL1-192 clone, because increased binding of its specific ligand PtC correlates with enhanced BCR signaling and cell proliferation in the spleen and lymph nodes, which correlates with observations in patients [33].

While αSlamf6 treatment causes a significant loss of leukemic cells in the blood and spleen of TCL1-192-bearing SCID mice, as well as LMP2A/λMyc bearing Rag1^−/−^ mice, both tumor cells remain in the peritoneal cavity of the recipient animals. The Chiorazzi lab discovered that the tumor microenvironments, *e.g.* spleen vs. peritoneal cavity, influences the way TCL1-192 cells respond to antigenic stimuli and BCR signaling [33]. It is therefore plausible that the αSlamf6 antibody does not affect peritoneal TCL1-192 cells, because of the protective microenvironment and altered signaling of the leukemic cells in the peritoneal cavity. In support of this concept is that administering Rituximab (αCD20) does not remove B220^+^CD5^+^ B1a cells from the peritoneal cavity, while it is very efficient in the killing of B cells in most tissues [42]. Impaired Fc-receptor functions have been implicated [42]. Similarly, CD5^+^ B1 cells respond differently to BCR stimuli in the peritoneum of WT mice than that in the spleen due to expression of the Src kinase Lck, which renders peritoneal B1 cells hypo-responsive [50–52]. Interestingly, most human CLL cells express Lck albeit at varying levels [53]. Importantly, the outcomes of several studies indicate that some CLL patients have massive ascites, suggesting that the tumor cells in the peritoneal cavity may not respond to some treatment protocols [54].

We find that in the blood two sub-populations of mouse TCL1-192 CLL cells exist, *i.e.* IgM^hi^ / CXCR4^hi^ / B220^hi^CD5^hi^ and IgM^low^ / CXCR4^low^ / B220^int^CD5^int^ TCL1-192 cells. Coelho et al. [41] suggest that IgM^low^ patient-derived CLL cells have just entered the circulation, as the low levels of BCR are caused by antigenic exposure within tissues. By contrast, cells that have been in circulation longer display a higher BCR expression [41]. A plausible interpretation of our observations is therefore that IgM^low^ / CXCR4^low^ / B220^int^CD5^int^ TCL1-192 cells are entering into the circulation from the peritoneal cavity, spleen or other secondary lymphoid organs. Administering αSlamf6 eliminates this IgM^low^ / CXCR4^low^ / B220^int^CD5^int^ TCL1 subset in the blood. While half of the IgM^hi^ / CXCR4^hi^ / B220^hi^CD5^hi^ cells are in the process of apoptosis as judged by the Annexin-V staining, the other half of this sub-population is possibly on its way to migrating to other tissues for antigenic stimulation [41]. Alternatively, the pro-apoptotic B220^hi^CD5^hi^ population may already have undergone cell membrane damage, thus not allowing proper binding of the antibody for efficient targeting. Expression levels of CXCR4 support the concept, as this chemokine receptor is not only a migration marker, but is also involved in apoptosis of CLL and acute myeloid leukemia cells [55, 56].

Administering a combination of αSlamf6 and ibrutinib eliminated TCL1-192 tumors in the recipient SCID mice due to a synergistic effect, which was greater than caused by each agent alone. The most likely explanation is that ibrutinib down-regulates BCR signaling and causes apoptosis of the tumor cells within the peritoneum and elsewhere in the body. Second, as ibrutinib is known to move CLL cells out of their various niches into the circulation and removal of leukemic cells from the peritoneal cavity could have resulted in this synergistic reduction of the overall tumor burden. Our use of αhSLAMF6 in *in vitro* and *in vivo* systems using CLL cell lines support the potential of the antibody as a therapeutic target and deserves further investigation using primary CLL cells. Thus, the outcomes of this study suggest that αSLAMF6 and ibrutinib should be considered as a combination therapy for CLL and possibly other SLAMF6 expressing B cell tumors.

## MATERIALS AND METHODS

### Mice

CB17 SCID and Rag2^−/−^γc^−/−^ mice from Taconic (Hudson, NY) and Rag1−/− mice from the Jackson Laboratory are maintained under specific pathogen-free conditions at the Beth Israel Deaconess Medical Center (BIDMC) animal facility. Experiments were performed according to the guidelines of the Institutional Animal Care and Use Committee (IACUC) at BIDMC.

### CLL cells

Peripheral blood samples were obtained after informed consent from CLL patients at BIDMC, Department of Hematology/Oncology. Patient consent for samples used in this study was obtained in accordance with the Declaration of Helsinki on protocols that were approved by the Institutional Review Board at BIDMC. Peripheral blood mononuclear cells (PBMCs) were isolated using lymphocyte separation medium (Corning, Manassas, VA).

The human CLL MEC-1 cell line [36] was a gift from Dr. Silvia Deaglio (University of Turin, Italy). Cells were cultured in RPMI 1640 medium, as described [57]. The human OSU-CLL cell line [37], which was generously donated by Dr. John C. Byrd (Ohio State University), was cultured as described.

The leukemic TCL1-192 clone (B6xC3H) was generated as described [33]; it's BCR recognizes phosphatidylcholine (PtC) with a single IgHV-D-J rearrangement.

### Flow cytometry

PBMCs from CLL patients, the MEC-1 and OSU-CLL cell lines were stained with: PE, FITC or APC conjugated anti-human monoclonal antibodies were purchased from Biolegend (San Diego, CA): CD3 (HIT3a), CD19 (HIB 19), CD5 (UCHT2), SLAMF1 (A12), SLAMF2 (BJ40), SLAMF3 (Hly-9.1.25), SLAMF4 (C1.7), SLAMF5 (CD84.1.21), SLAMF6 (NT-7), SLAMF7 (162.1), PE-anti-mouse mIgG1 (MOPC-21), anti-mIgG2a (MOPC-173) and anti-mIgG2b isotype controls (MPC-11). Anti-human SLAMF8 (250014) was from R&D Systems (Minneapolis, MN). SLAMF surface expression on CLL cells from 57 patients was determined with antibodies provided by the “Ninth International Workshop on Leukocyte Antigens” [22].

Dead cells were excluded by DAPI staining and cell count per μl of blood was determined by CountBright absolute counting beads (Life Technologies, Carlsbad, CA).

PE, FITC, APC, APC/Cy7, PerCP/Cy5.5, PE/Cy7 or Pacific blue conjugated anti-mouse antibodies were purchased from Biolegend: B220 (RA3-6B2), Slamf1 (TC15-12F12.2), Slamf2 (HM48-1), Slamf3 (Ly9ab3), Slamf5 (mCD84.7), Slamf6 (330-AJ), IgM (RMM-1) and CXCR4 (L276F12). Anti-mouse CD5 (53-7.3) and Slamf4 (eBio244F4) antibodies were purchased from eBioscience (San Diego, CA). Anti-mouse Slamf7 (520914) antibody was purchased from R&D Systems (Minneapolis, MN).

For intracellular staining with the BD Cytofix/Cytoperm Kit, the following antibodies were used: pZAP70/pSyk(Y319/Y352) (n3kobu5) and pBtk(Y551/Y511) (M4G3LN) from eBioscience and pPLCγ2(Y759) (K86-689.37) from BD Biosciences (San Jose, CA). The procedure described in the manufacturer's manual was followed.

The Annexin-V Apoptosis Kit was purchased from eBioscience.

The cells were acquired on a BD LSRII flow cytometer and data analysis was performed using FlowJo software (Tree Star Inc., Ashland, OR).

### LMP2A/λMyc tumor cells

Peripheral lymph node tumor cells from LMP2A/λMyc (Tg6/λMyc) mice [35] were *i.p.* injected into Rag1^−/−^ mice.

### Histology

Liver sections were fixed in 10% formalin and stained with H&E at the BIDMC histology core.

### Reagents

The mouse monoclonal αSlamf6 (clone 13G3) hybridoma was generated from spleens of Slamf6^−/−^ mice immunized with WT thymocytes [26]. Hybridomas were used to produce IgG2a by Harlan Laboratories (South Easton, MA). Mouse IgG2a (C1.18) isotype control was purchased from BioXcell, Inc. (West Lebanon, NH). Mouse anti-human SLAMF6 antibody (994.1) was obtained from ARCA Biopharma (Westminster, CO).

F(ab')_2_ goat anti-mouse IgG was purchased from Jackson Immunoresearch.

The BTK inhibitor, ibrutinib, was purchased from ChemieTek (Indianapolis, IN). Ibrutinib was dissolved in DMSO and prepared as described elsewhere [49].

### *In vitro* culturing of OSU-CLL

5 × 10^4^ OSU-CLL cells were seeded in 96-well flat bottom plate. Relevant wells received 50 μg/ml Fc-crosslinker, 10 μg/ml αSLAMF6 or 0.5 μM ibrutinib. Plate was incubated for 72 hours at 37C and 5% CO_2_. Viability was measured by Annexin-V/PI staining.

### Injection of MEC-1 in [Rag x γc]^−/−^ mice

10^7^ MEC-1 cells were injected subcutaneously into [Rag x γc]^−/−^ (Taconic) mice and monitored as previously described [44].

### Statistics

Statistical analyses were calculated using GraphPad Prism software (GraphPad, La Jolla, CA). The Student t test or 2-way ANOVA was used to compare groups; results are represented as mean ± SD. P < 0.05 was considered significant.

## SUPPLEMENTARY FIGURES


