# Supplementary material for: A combination of an anti-SLAMF6 antibody and ibrutinib efficiently abrogates expansion of chronic lymphocytic leukemia cells
Source: Oncotarget. 2016 Mar 25;7(18):26346–60. doi: 10.18632/oncotarget.8378 (PMC5041984; doi:10.18632/oncotarget.8378)
Supplement: Supplementary file 1 [file oncotarget-07-26346-s001.pdf]

## SUPPLEMENTARY FIGURES

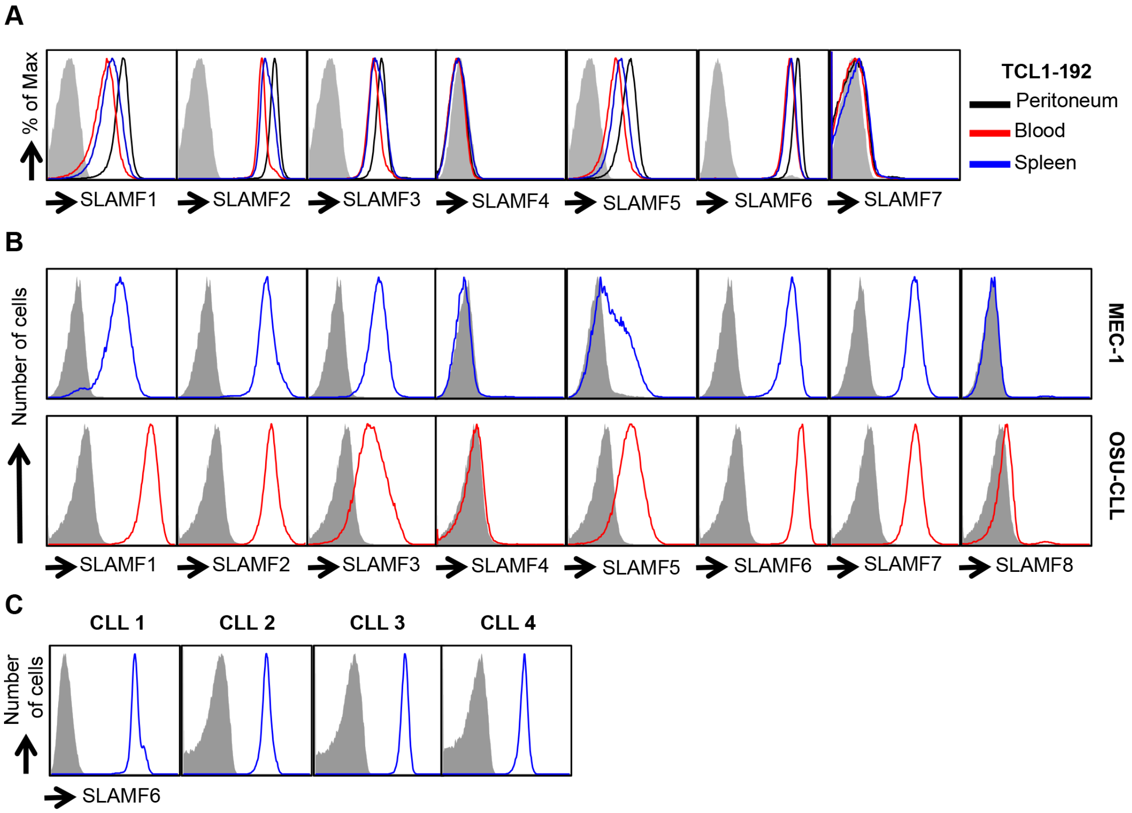


**Supplementary Figure S1: Expression of SLAMF receptors on the surface of CLL cells. A.** Representative histograms of Slamf receptor expression on the surface of TCL1-192 cells. Four weeks after *i.p.* injection into SCID mice (0.5 × $10^6$ cells/mouse) TCL1-192 cells were isolated from the peritoneal cavity (referred to as peritoneum throughout the figures), blood or spleen. Gray bars indicate isotype controls or FMO (Fluorescence minus one) in all histograms. TCL1-192 cells in the peritoneal cavity also express the highest levels of Slamf1 (MFI P: 3952, B: 1218, S:1827), Slamf2 (MFI P:22850, B:8906, S:13810), Slamf3 (MFI P:3637, B:2139, S:2852) and Slamf5 (MFI P:1488, B:553, S:780). **B.** Human CLL cell lines MEC-1 and OSU-CLL were cultured *in vitro*, and expression of SLAMF receptors was determined by flow cytometry. **C.** PBMCs were obtained from the blood of 4 CLL patients. SLAMF6 expression was determined on $CD3^-/CD19^+CD5^+$ CLL cells using flow cytometry.

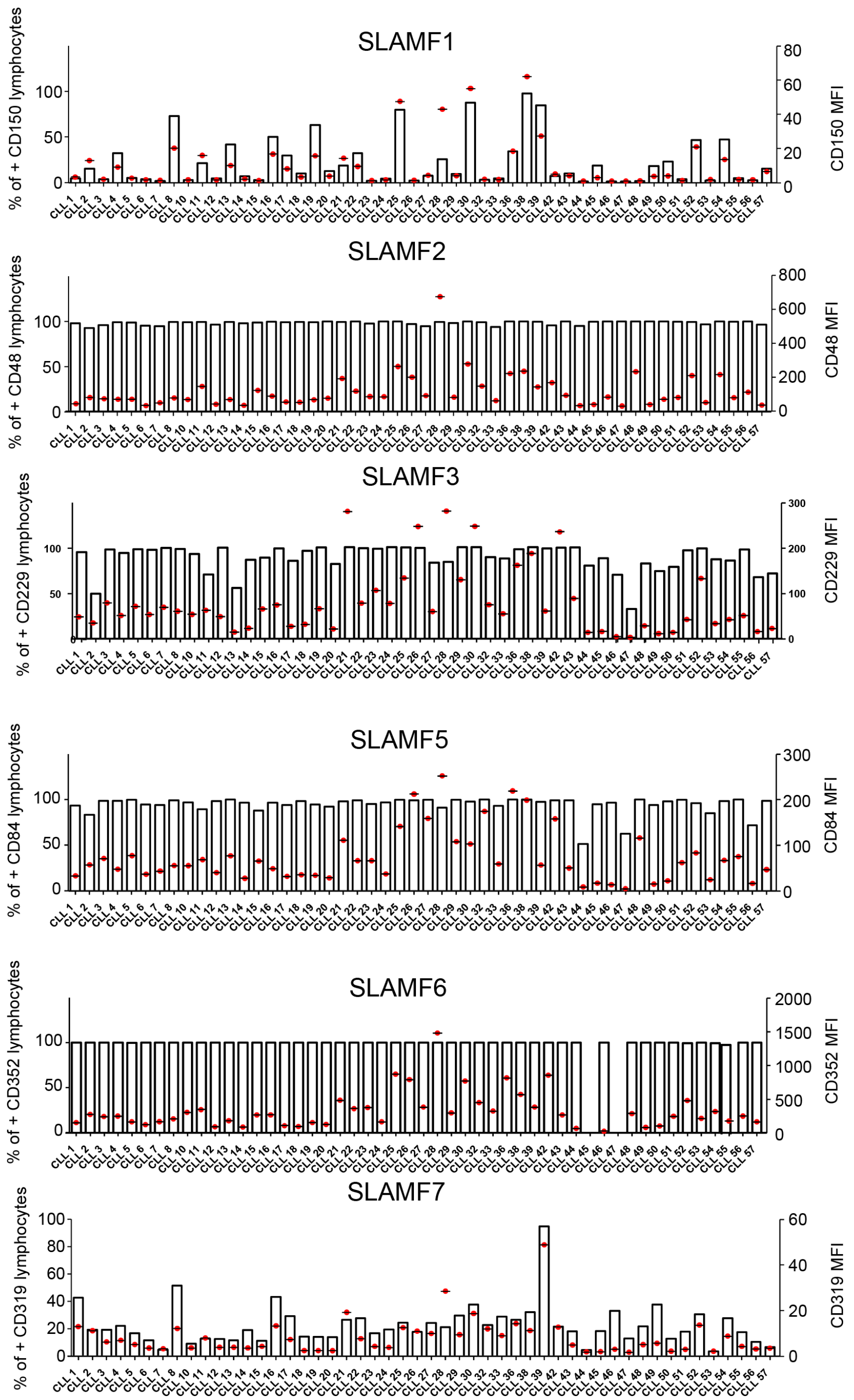


**Supplementary Figure S2: Expression of SLAMF receptors on the surface of CLL cells isolated from 57 patients.** Cell surface expression of SLAMF molecules from PBMCs of 57 CLL patients was measured using flow cytometry. The left y-axis represents the percentage of expression and the right y-axis represents the MFI values for each molecule. Representative flow cytometry plot showing the overlay of B220$^{+}$CD5$^{+}$ cells from the peritoneum, blood and spleen.

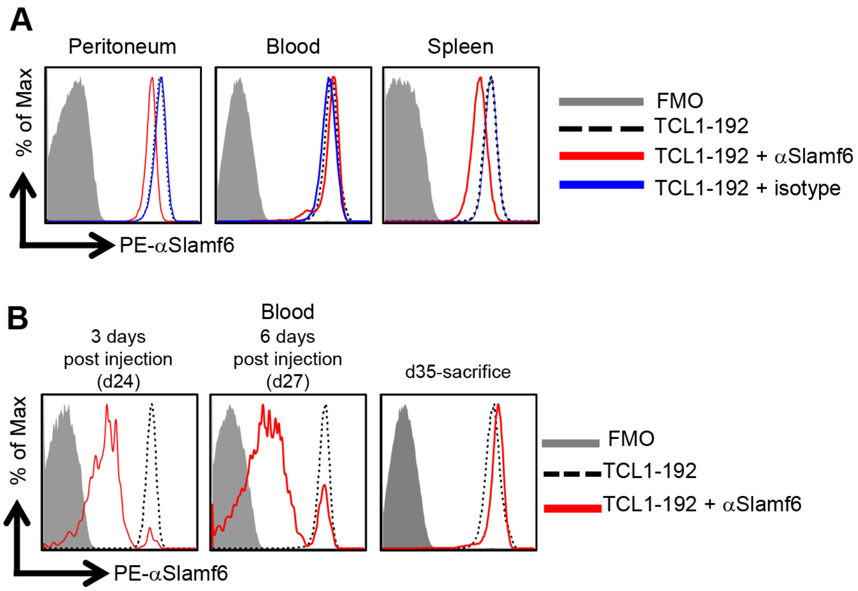


**Supplementary Figure S3: Slamf6 expression on the surface of TCL1-192 cells after injection of αSlamf6. A.** Expression of Slamf6 on TCL1-192 cells from non-injected, αSlamf6 or isotype control injected mice was measured. Cells in the spleen, blood and peritoneal cavity at d28 (from experiment outlined in Figure 1A) were stained for Slamf6 receptor availability by PE-conjugated anti-mouse Slamf6 antibody (details in Materials and Methods), and determined by flow cytometry on B220$^{+}$CD5$^{+}$ cells. Histograms are representative from each group. **B.** Occupancy of the Slamf6 receptor by the injected αSlamf6 on the surface of B220$^{+}$CD5$^{+}$ cells in the blood was assessed 3 and 6 days after the first αSlamf6 injection (days 24 and 27) and at d35. Representative histograms of *in vitro* staining with a PE-conjugated αSlamf6 FACS antibody comparing unoccupied Slamf6 receptor expression in αSlamf6 injected and non-injected groups.

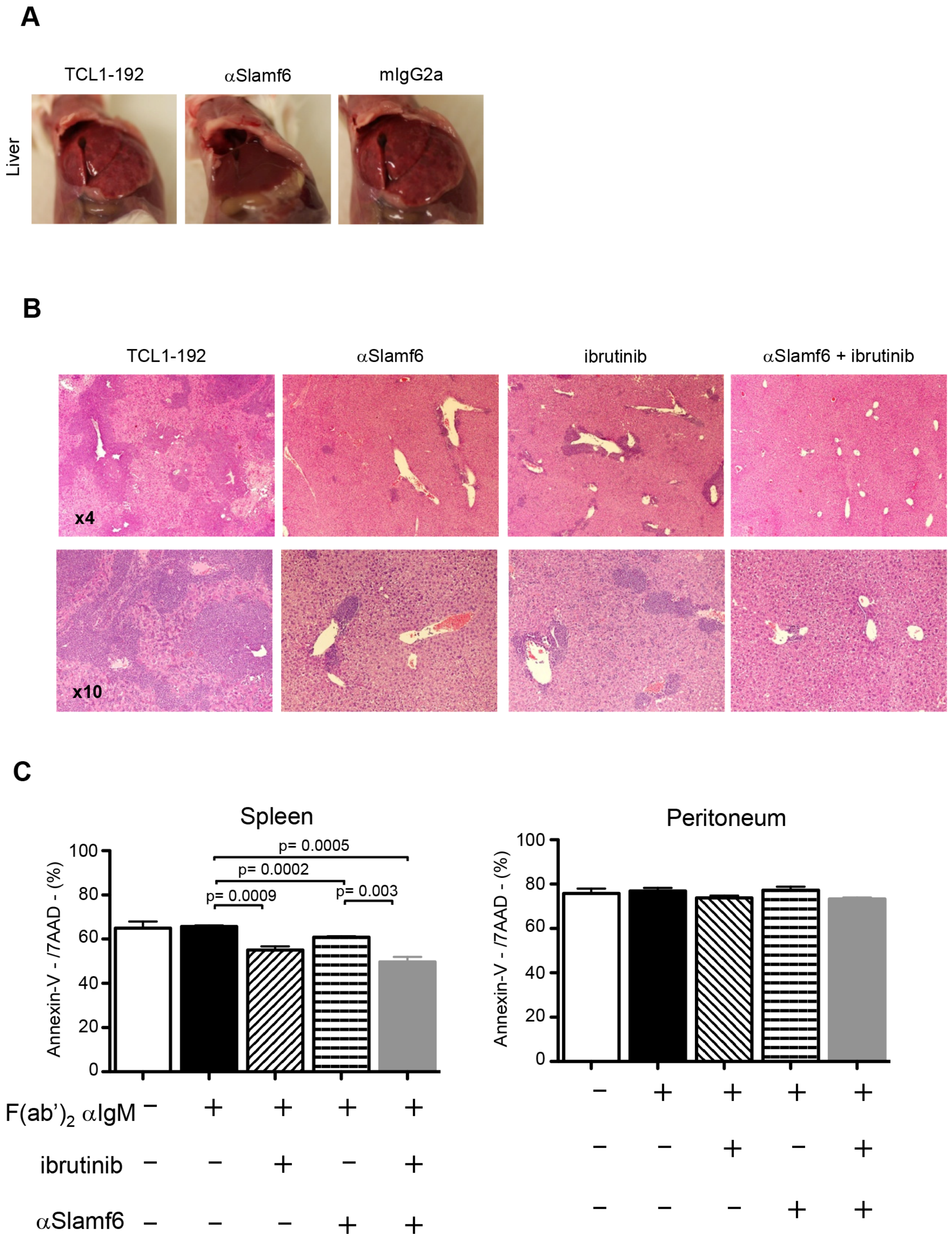


**Supplementary Figure S4: Anti-Slamf6 + BTK inhibitor, ibrutinib, has synergistic effect on liver and in *in vitro* apoptosis of TCL1-192 cells. A.** Representative figure indicating TCL1-192 leukemic infiltration in the liver of SCID mice on d35, as indicated in Figure 2B. **B.** Representative H&E staining of liver from experiment in Figure 6. Double treated mice show no sign of leukemic infiltration compared to single treated mice. **C.** *In vitro* stimulation and treatment of splenic TCL1-192 cells in the presence of both αSlamf6 and ibrutinib, results in significantly higher Annexin-V+ apoptotic cells compared to either alone (Left Panel). Viability of peritoneal TCL1-192 cells were not affected from treatment with either regimen.

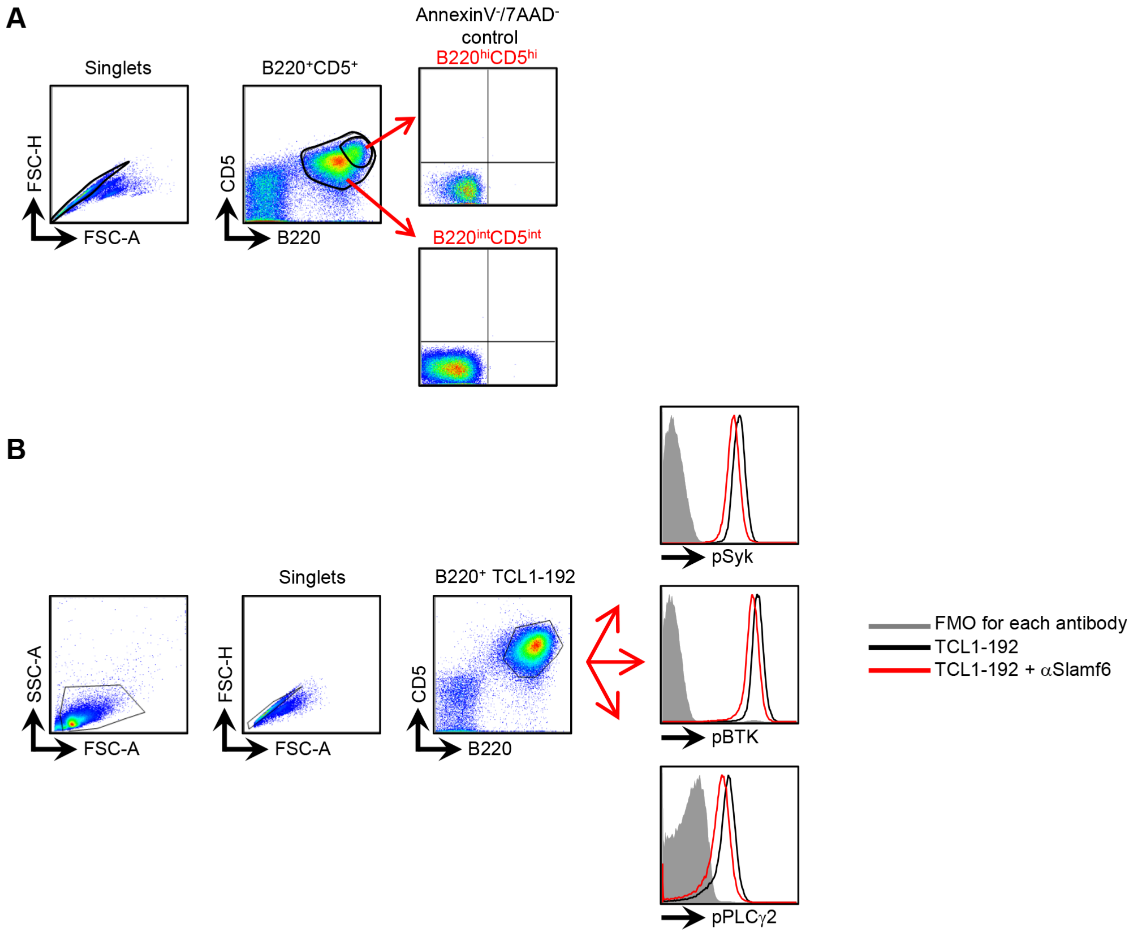


**Supplementary Figure S5: Gating strategy of AnnexinV/PI and phosphoflow staining. A.** Representative plots of B220+CD5+ gating in blood (Figure 5A). Corresponding B220$^{hi}$CD5$^{hi}$ or B220$^{int}$CD5$^{int}$ populations were gated based on AnnexinV/PI negative control group. **B.** Representative gating for pSyk, pBtk and pPLCγ2 antibodies (Figure 5C-F). Singlet cells were determined and B220+CD5+ TCL1-192 cells were gated. Fluorescence minus one (FMO) was used as a negative control for each antibody staining.
